# I had a dream

**DOI:** 10.1002/ccs3.12064

**Published:** 2025-01-13

**Authors:** Bernard Perbal

**Affiliations:** ^1^ International CCN Society Nice France

**Keywords:** editor in chief, editorial board, Journal of Cell Communication and Signaling

## Abstract

Expressing hopes and desires is an essential part of communication, and communication is the major pillar of the Journal of Cell Communication and Signaling. There comes the time of giving the responsibility of leading JCCS to a new editor in chief and I am taking this opportunity to comment on a few recent milestones and future of the journal which I created in 2007, after I had created “Cell Communication and Signaling” 20 years ago (1), one of the first open access journals.

## Graphical abstract

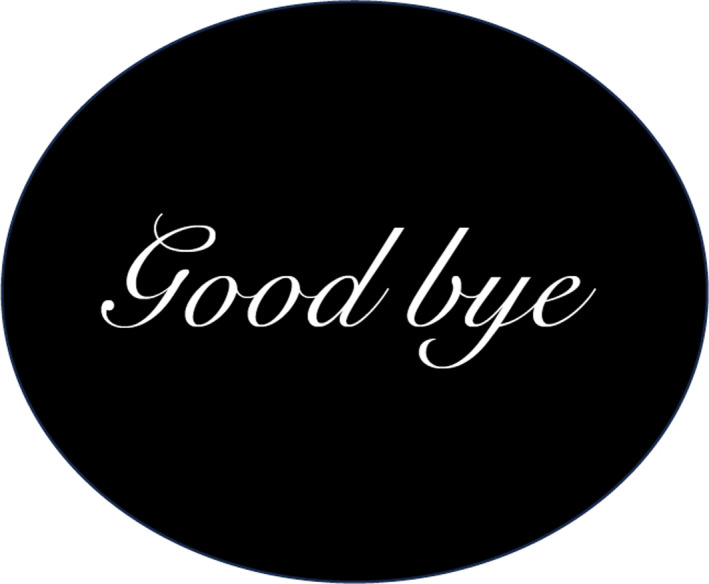

Back in August 2023, after the move of JCCS from Springer to Wiley had been made official, I committed myself to remain editor in chief until the end of 2024 in order to ensure that the transition from Springer would be as smooth as possible.

The scientific context that had guided me to operate the move to a new publisher has been discussed in a previous editorial.^1^

The JCCS editorial board members, who convened during the 2022 Nice international workshop on the CCN family of genes, fully agreed that our Journal needed “fresh blood and ideas” to reinforce its scientific recognition.

Stepping up and establishing JCCS in the highly competitive realm of scientific publishing was a dream that was required to confront a great challenge.

The support that I got from Wiley, thanks to former CEO Brian Napack, was an extremely positive stimulus.

My dream might come true.

We just needed to follow the track opened by the Wiley executive leadership team “*We clear the way for seekers of knowledge, illuminating the path forward for research and education, tearing down barriers to society's advancement, and giving seekers the help they need to turn their steps into strides*”.^2^

Dedicated seekers and educators we are, through our visions, commitment, and contributions to the communication of sciences advances^3^ that must benefit humankind.

Time flies and there we are.

Facing a “universe” of stimulating challenges.

## 1 JCCS IN 2024

Joining Wiley was expected to boost our publishing activity and expand our readership.

The successful transition was only possible thanks to the commitment and continued support of a reshaped board of editorial members. It was also dependent upon the expected professionalism of the team in charge of JCCS integration and expansion in the Wiley organization.

The newly organized JCCS board includes an executive board constituted by four executive editors (ExE) and the editor in chief (EiC). The triage of submissions which need to comply with the instructions to authors is presently performed by the EiC who hands over the adequate submissions to the qualified ExEs who dispatch the manuscripts to associate editors of the board.

Because of internal constraints, we could not keep the original proposed organization in which two distinct layers of associate editors and editors were supposed to interact with each other. I believe that this point should be reconsidered by the Wiley administrators.

This workflow is unusual at Wiley and we had a fair load of discussions to get it accepted by the JCCS administration. It has not been easy, but presently, it turns out to be efficient even though it still needs some adaptation.

I wish to express here my deepest thanks to the four executive editors and to Annick (administrative assistant and coordinator at that time) for their support and help in getting the submission process to move on smoothly.

My acknowledgments also go to the associate editors and reviewers for their participation. Without their involvement and support, JCCS would not be successful.

The sustained commitment of all the editorial board members has been instrumental to JCCS maintaining its position and becoming recognized as a leader in the field of Cell Communication and Signaling.

## 2 THE FUTURE

In spite of the considerable amounts of efforts requested from all those who participated actively in the challenges that we faced in order to maintain a satisfactory publication rate, we can consider that JCCS had a successful year and we have hopes that it will maintain its momentum by introducing a new editorial board in 2025.

I had accepted to remain editor in chief of JCCS for only one more year to facilitate the transition from Springer to Wiley. This has been extremely demanding, leaving only an insufficient time to manage my other activities and private life.

My present hopes and desires for JCCS can be summarized in my wish to see the journal survive and fight to get afloat at a good position among the best ones in the 4.3 millions of yearly published scientific communications (Table 1).

**TABLE 1:** 

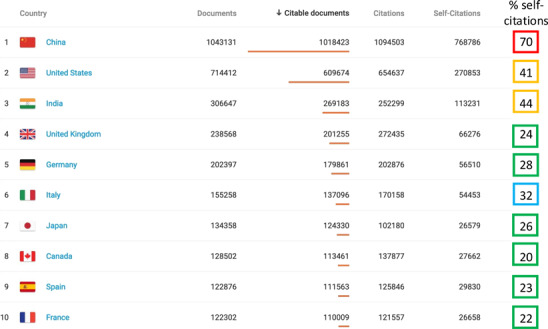

*Note*: The table shows the 2023 country ranking for scientific journals publishing, as modified from the presented data in Scimago Journal and Country rank, available at: https://www.scimagojr.com/countryrank.php?order=itp&ord=desc&year=2023.

I previously expressed my sincere feeling that the journal requires the input of a new and younger management to reinforce its position in a very competitive publishing realm.

I am now proud to inform our readers and authors that Wiley has accepted my proposition to nominate our talented colleague and friend Professor Brahim Chaqour as the next editor in chief of JCCS. I know Brahim as a scientifically minded, well‐published, thoughtful scientist and dear friend who is well suited to take up the challenges and carry the torch to help maintain the competitive expansion of JCCS.

As the founding editor of JCCS, I will continue to provide my support and insights to keep the journal alive and growing. Let us celebrate this transition and the continued success of JCCS at our next CCN workshop and ARBIOCOM meeting.

The time for the termination of this contract with Wiley has come and I unfortunately do not have enough space to take this opportunity and sincerely express my heartful recognition to all of you who were always on my side to offer their support over all these sometimes bumpy 20 past years of publishing.

From the inception of JCCS to the present time, I cannot skip mentioning the honor and pleasure that I had to share my enthusiasm, ideas, and fears with Peter Butler, Alison Mitchell, William Curtis, Jamie Hutchins, Jaco Flipsen, Meran Lloyd‐Owen, Hermine Vloemans, my scientific visions with Lester Lau and my four colleagues of the Executive board, Havard Attramadal, Brahim Chaqour, Kathryn Meier, and Ralf Weiskirchen. I also acknowledge Andrew Leask's contributions in the early JCCS period.

Particular thanks are due to my sincere friend and collaborator Herman Yeger whose constant support, criticisms, and suggestions were at the root of my extraordinary journey in the world of “recent” scientific publishing, ICCNS and ARBIOCOM.

Finally, I could not close up this farewell editorial without thanking again my dear wife Annick for sharing with me the good the bad and the ugly of being the founder and editor in chief of a few journals since my first high school time…this is another story.

It is now time to say “Good bye everyone”.

## CONFLICT OF INTEREST STATEMENT

The author declares no conflicts of interest.

## Data Availability

Data sharing is not applicable to this article as no new data were created or analyzed in this study.
